# Statistical deconvolution of enthalpic energetic contributions to MHC-peptide binding affinity

**DOI:** 10.1186/1472-6807-6-5

**Published:** 2006-03-20

**Authors:** Matthew N Davies, Channa K Hattotuwagama, David S Moss, Michael GB Drew, Darren R Flower

**Affiliations:** 1 Edward Jenner Institute for Vaccine Research, Compton, Newbury, RG20 7NN, UK; 2 School of Crystallography, Birkbeck College, London WC1E 7HX, UK; 3 Structural and Computational Chemistry Group, University of Reading, Reading RG6 6AH, UK

## Abstract

**Background:**

MHC Class I molecules present antigenic peptides to cytotoxic T cells, which forms an integral part of the adaptive immune response. Peptides are bound within a groove formed by the MHC heavy chain. Previous approaches to MHC Class I-peptide binding prediction have largely concentrated on the peptide anchor residues located at the P2 and C-terminus positions.

**Results:**

A large dataset comprising MHC-peptide structural complexes was created by re-modelling pre-determined x-ray crystallographic structures. Static energetic analysis, following energy minimisation, was performed on the dataset in order to characterise interactions between bound peptides and the MHC Class I molecule, partitioning the interactions within the groove into van der Waals, electrostatic and total non-bonded energy contributions.

**Conclusion:**

The QSAR techniques of Genetic Function Approximation (GFA) and Genetic Partial Least Squares (G/PLS) algorithms were used to identify key interactions between the two molecules by comparing the calculated energy values with experimentally-determined BL_50 _data. Although the peptide termini binding interactions help ensure the stability of the MHC Class I-peptide complex, the central region of the peptide is also important in defining the specificity of the interaction. As thermodynamic studies indicate that peptide association and dissociation may be driven entropically, it may be necessary to incorporate entropic contributions into future calculations.

## Background

Major Histocompatibility Complex (MHC) molecules play a central role in the adaptive immune system, forming a complex with foreign antigenic peptides and displaying them on the cell surface for inspection by receptors of cytotoxic and helper T cells. In consort with the T cell receptor, the repertoire of MHC-peptide complexes shapes the specificity of the T cell response. Two different types of MHC molecules – Class I and Class II – are recognized by distinct sets of T-cells: CD8+ and CD4+ respectively. MHC Class I molecules, which predominantly present antigenic material derived from the cytosol, are composed of an α heavy chain, a light chain (β_2_-microglobulin or β_2_m) and a peptide between 8–15 amino acids in length. The peptide is bound within a groove formed by the MHC heavy chain.

Analysis of MHC Class I-peptide binding interactions together with specific peptide-MHC crystal structures reveals several common trends. A conserved cluster of tyrosine residues are present at the N terminus of the peptide while a different set of MHC Class I residues form a series of hydrogen bonds and ionic interactions with the C terminus and peptide backbone. Previous attempts to predict potential epitopes have concentrated on binding motifs. Such motifs greatly emphasize so-called 'anchor' residues at peptide position P2 and the peptide C-terminus, which occupy the B and F pocket of the MHC Class I binding groove respectively [1,2]. The B pocket is composed of eight α heavy chain residues (9, 24, 45, 66, 67, 70 and 99) and the F pocket is composed of four (77, 80, 81 and 116). Previous analysis of HLA-A*0201 [3] found that residues such as leucine, isoleucine, valine and methionine are favoured for the P2 position while the optimal residues for the C-terminus are leucine and valine (although methionine, isoleucine, alanine and threonine can also occupy this position). In some cases, contributions made by the peptide residues at positions 1, 3, 6 and 7 can compensate for the presence of unfavoured residues at the P2 and C-terminal positions). In an attempt to discover novel T cell epitopes, many motif-based techniques have been used to predict the affinity of MHC-peptide interactions. Diverse methods, such as EpiVax [4], Artificial Neural Networks [5], Hidden Markov Models [6], Support Vector Machines [7] and Profiles [8], have all been used to create T-cell epitope prediction programs. Typically, prediction techniques have been calibrated by using experimentally-determined IC_50 _and BL_50 _data [9], at least indirectly. Purely motif-based analysis of binding, however, may be too simplistic to accurately detect the effect of non-anchor residues within the groove. This is because changes in the peptide sequence may introduce subtle structural variations into the binding which can greatly influence peptide binding affinity [10]. These interactions can be partially accounted for by the additive effect of the binding. The calculation of the additive effect is based upon the method first introduced by Free and Wilson whereby each substitute makes additive and constant contributions to biological activity regardless of substituent variation. This assumes that peptide binding affinity can be decomposed into the sum of contributions from residues at each position and the interaction between those residues. The QSAR (Quantitative Structure-Activity Relationship)-based additive technique [10-16] is based on a more sophisticated approach than motifs and uses additive technique in the calculation. The additive QSAR model therefore accounts for the sum of amino acid interactions, the sum of adjacent peptide side chain interactions, and the sum of every second side chain interaction, and so on.

A possible way to full incorporate the additive effects into the prediction is to derive the affinities based on structural data. The COMBINE (COMparative BINding Energy) method does this by creating a model which estimates binding free energy differences based on energy-minimised structures of ligand-receptor complexes [17-19]. The model is fitted to a set of experimental binding parameters and is used to predict the affinity of novel ligands. This technique assumes that the free energy of binding correlates with a subset of energy components determined from the structure. In this paper, a technique similar to COMBINE is developed which incorporates both structural and data-driven methods in order to create a novel method for the analysis of peptide-MHC binding. This new method seeks to better understand the atomic interactions that determine binding affinities: static energy analysis calculations combined with QSAR analysis are used to calculate the relative contribution of interactions between the MHC Class I allele A*0201 and bound peptide residues, thus generating an accurate and predictive system for the A2 supertype.

## Results

QSAR analysis was carried out on three individual models: total/electrostatic/van der Waals energy descriptors; electrostatic/van der Waals energy descriptors and total non-bonded energy descriptors. The matrix consisted of 118 peptide rows and 4860 descriptor (energy, electrostatic and van der Waals) columns. To make this analysis tractable, any columns consisting entirely of values of ~0 kcal mol^-1 ^(signifying that no contributions were made between the peptide and binding site) were omitted from the dataset, yielding a final matrix of 1504 descriptor columns (454 total energy descriptors, 331 electrostatic energy descriptors and 719 van der Waals energy descriptors). The resulting regression equation modeled the binding affinities of a training set of 83 peptides and was used to predict the binding affinities of an independent test set of 35 peptides.

The GFA and G/PLS algorithms were used to select the optimum number of descriptors for use in the regression analysis. Although the methods are similar, the application of G/PLS often allows the construction of larger QSAR equations while still avoiding over-fitting and eliminating most variables. Within the *Cerius*^*2 *^software, the crucial variable is the initial number of descriptors chosen in the regression equation although this can be varied during the process by including a mutation probability for increasing or decreasing the equation length. The statistical indices for the best GFA and G/PLS algorithms for all 3 models are shown in Table 1. For all the QSAR models, the number of terms in the initial and final equations was varied between 15–20 descriptors. It was found that raising or lowering the number of descriptors outside of this range caused a steady increase in the PRESS value and a lowering of the r^2^. Models were created with 10, 25 and 30 descriptors and all exhibited a decrease in the quality of the model. The GFA Total + Electrostatic + VDW model is shown in Table 2 as example. It is likely that the use of too many descriptors or too few will cause overfitting or underfitting of the model respectively. 15–20 descriptors may represent a range where there is sufficient information to build a model without overemphasising specific interactions that do not represent general trends within the dataset. The best model was chosen based on both the r^2 ^value of the test set and also on the (CV)r^2 ^value from the training set. All selected models had the highest available test set r^2 ^value, except for the G/PLS Electrostatic + VDW model where the 15 descriptor model was chosen as it has a (CV)r^2 ^value significantly higher than the 16 descriptor model. Marked equations had a few anomalous outliers removed to consolidate the model.

Further analysis was also carried out limiting the potential descriptors to those amino acids comprising previously-defined MHC anchor pockets: P2 and the C terminus [1]. In that model, a total of 138 residues were used (46 energy terms, 46 electrostatic terms and 46 van der Waals terms). Despite the reduced population size, the optimal number of descriptors still occurred within the 10–20 descriptor range (see Table 3). The reduced number of descriptors generally seems to decrease the predictivity of the models significantly. However, one exception (the G/PLS Total + Electrostatic + VDW model) did produce a test set r^2 ^value of 0.711, which is higher than any model produced using the full range of descriptors. The GFA and G/PLS Total models produced extremely poor results with a test set r^2 ^value of 0.044 and 0.263 respectively. The best individual models for both the GFA and G/PLS algorithms taken from both Tables 1 and 3 are presented in Table 4 and 5 respectively. Descriptor sets taken from the best models generated by the GFA and G/PLS calculations were used to validate the QSAR analysis using the PLS module in SYBYL 6.9. The predictivity of GFA and G/PLS models compares well with the PLS method, indicating that the predictive ability of the models is consistent in terms of descriptor choice, despite differences in the form of the predictive equation in each case.

**Figure 1:**
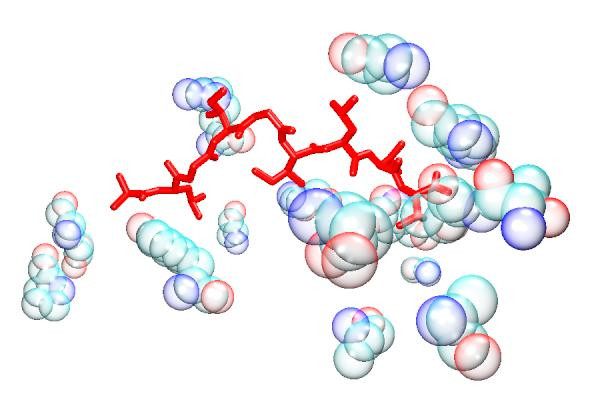
**GFA Total + Electrostatic + VDW model**. The bound peptide (red) is surrounded by the descriptor peptides (bubble opaque). Despite the apparent distance in some instances, it is established that long range interactions can be as important to the specificity of the binding as short range ones.

**Figure 2:**
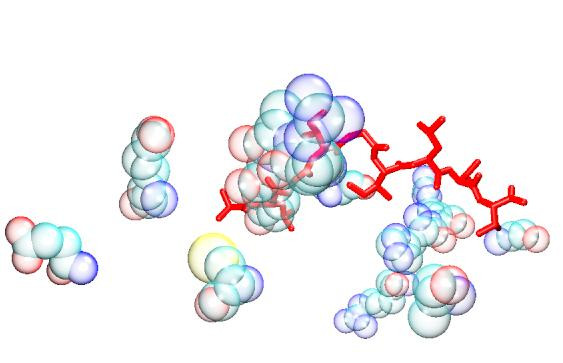
**G/PLS Total + Electrostatic + VDW model**. The bound peptide (red) is surrounded by the descriptor peptides (bubble opaque). Despite the apparent distance in some instances, it is established that long range interactions can be as important to the specificity of the binding as short range ones.

**Figure 3:**
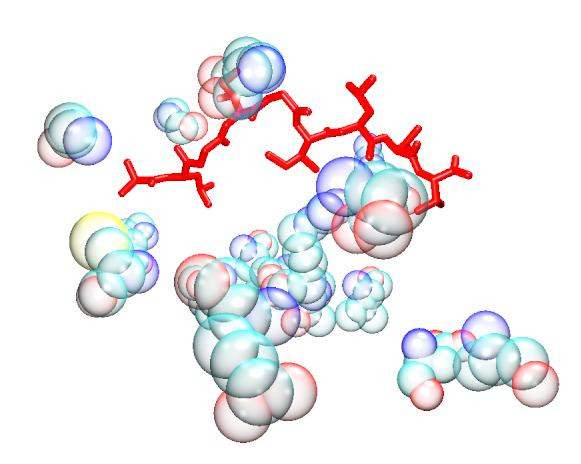
**GFA Electrostatic + VDW model**. The bound peptide (red) is surrounded by the descriptor peptides (bubble opaque). Despite the apparent distance in some instances, it is established that long range interactions can be as important to the specificity of the binding as short range ones.

**Figure 4:**
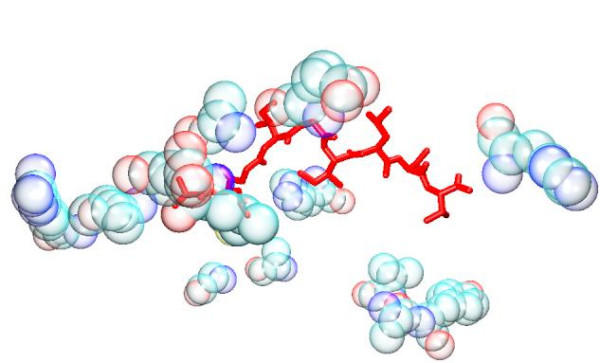
**G/PLS Electrostatic + VDW model**. The bound peptide (red) is surrounded by the descriptor peptides (bubble opaque). Despite the apparent distance in some instances, it is established that long range interactions can be as important to the specificity of the binding as short range ones.

**Figure 5:**
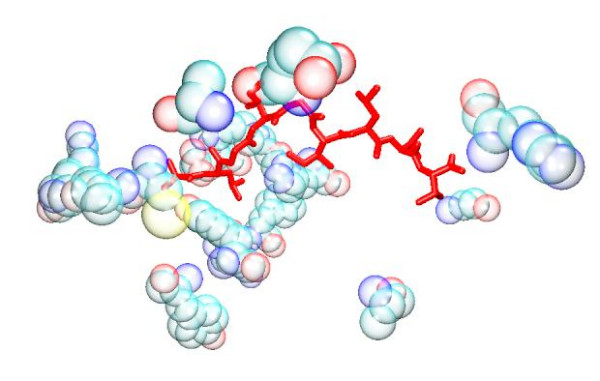
**GFA Total model**. The bound peptide (red) is surrounded by the descriptor peptides (bubble opaque). Despite the apparent distance in some instances, it is established that long range interactions can be as important to the specificity of the binding as short range ones.

**Figure 6:**
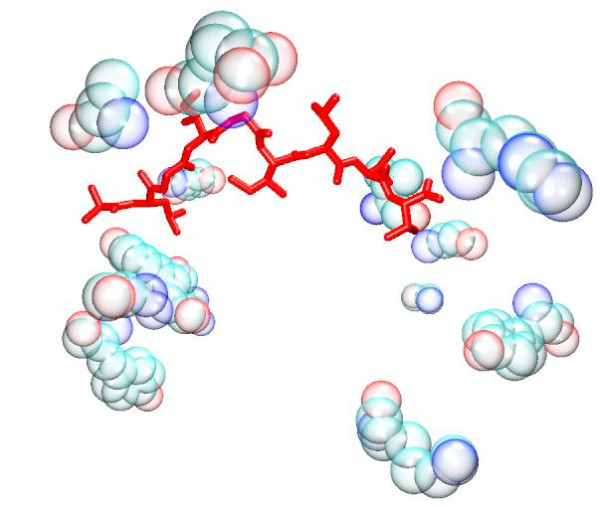
**G/PLS Total model**. The bound peptide (red) is surrounded by the descriptor peptides (bubble opaque). Despite the apparent distance in some instances, it is established that long range interactions can be as important to the specificity of the binding as short range ones.

Table 6 shows the residues that make up the descriptors for each of the six *Cerius*^*2 *^QSAR models presented in Table 3. Each model's residue distributions around the peptide are shown in Figures 1, 2, 3, 4, 5, 6. The selected coefficients are generated from those residues which make a consistent contribution over the entire training set. An overview of the QSAR models would suggest that although specific MHC-peptide interactions are difficult to identify, certain regions of the α heavy chain seem to relate to specific peptide residues. P2 is associated with the α60–70 region while the α160–170 region is associated with P2-P4. The α150–160 and α140–145 regions relate to P5 and P7 specifically. These associations may be fundamental to the binding and are maintained irrespective of the sequence of the peptide. In several cases, the MHC residues interact with the main chain atoms of the peptide and therefore make energetic contributions independent of the peptide sequence. Conversely, regions α1–4, α26–33, α46–58, α101–111 and α168–180 all make little or no energetic contribution due to their distance from the peptide residues. It is difficult to identify specific interactions from MHC residues, however, the following associations between specific MHC and peptide residues are all represented in three out of the six models: Met 5 interacting with P3, Asp 77 interacting with P9, Thr 143 interacting with P9, Val 152 interacting with P6 and Leu 156 interacting with P5. This might suggest that those five interactions all have an effect in determining the specificity of the interaction. Of the B pocket residues, Tyr 99 is the only residue to appear in two models while B pocket residues Ala 24, Met 45, Lys 66 and Tyr 116 only appear in one model each. Asp 77 appears in three models as is the only one of the four residues lining the F pocket which is used as a descriptor. Both Lys 66 and Asp 77 have been identified as forming conserved hydrogen bonds within the groove, respectively with the oxygen of the P2 residue and the nitrogen of the P9 residue [1]. Lys 66 is of particular interest as it has been identified as being a functional "hotspot" due to the formation of an ion pair with Glu 63 and an apparent dual role in both peptide binding and T cell receptor interaction [20-22]. In spite of this the residue is only used as a descriptor by G/PLS Total + Electrostatic + VDW model. The conserved bonds, Glu 63- P2 amide nitrogen and Tyr 99-P3 amide nitrogen, are not represented at all. Both residues, however, are used as descriptors in relation to P1 and in relation to P2 and P4 respectively in separate models. Previous work [10] has identified residues 9, 97, 114 and 116 as key to defining the A2-supertype, hence representative of vital points of interaction within the groove. They are poorly represented by the QSAR models, the only equation that incorporates one of them is the G/PLS Electrostatic + VDW model, where 116 interacts with P5. However, they do all occur within regions of the groove identified as important to the binding, again suggesting that it is regions rather than specific residues that determine the stability of the complex.

## Discussion

Overall, within our models, the distribution of the descriptors does not lend support to the view that burial of anchor residues, within defined cavities, are mainly responsible for stabilising the peptide within the groove. Rather it indicates a fairly even distribution of MHC residue interactions along the length of the peptide. Attempts to improve the QSAR model by focusing on the MHC residues associated with the anchor residues were largely unsuccessful (see Table 3 and Table 5), in spite of the predictive accuracy of the G/PLS Total + Electrostatics + VDW model. Despite the fact that the anchor models generate a similar number of descriptors to those of the full groove, focusing the interaction towards the peptide termini at the expense of the variable region generally reduces the algorithm's predictivity. It is possible that this reflects the comparative dominance of electrostatic interactions in determining binding affinity within both of the anchor pockets. Model predictivity also deteriorates after removal of each descriptor type compared to the full residue model, particularly in the case of models based on Total energy. Moreover, attempts to split the interaction energy into long range electrostatic and short range van der Waals forces also decreases correlation in the full residue model, suggesting that a single residue's total energy contribution is primarily enthalpic in character. It should be noted that several of the residues highlighted by the models are distant from the peptide (see Figures 1, 2, 3, 4, 5, 6), demonstrating the importance of long-range electrostatic interactions, which can be up to 8Å length, in stabilizing the complex. This could also be an effect of superadditivity correlation (SAC) [23] where correlation between variables between two sites does not imply that the two sites directly interact. Mutational data has indicated that long range non-causal interactions can be stronger in magnitude than causal correlations induced by direct interactions [24,25].

The Total energy models showed the highest correlation in the test set for both the GFA and G/PLS model although the (CV)r^2 ^value of the G/PLS model dropped sharply in the training set. Both models placed great emphasis on the conserved P1-P3 region of the MHC Class I groove at the expense of the central and C-termini regions. The key interaction of Asp 77 with the P9 residue was incorporated into both the GFA and G/PLS model, indicating that it may be a vital descriptor for a successful predictive algorithm. Both sets of models where the energetic contributions were broken down into the electrostatic and van der Waals contributions showed a lower degree of correlation (although the PRESS values for the Test Set compared favourably with the Total Energy Models). Building comparative models using the program SYBYL (see Table 4 and 5) produced a consistently lower level of correlation compared to the *Cerius*^*2 *^model but the apparent trends are comparable. The QSAR models also show a similar degree of correlation to models generated by the additive method [10] which produced a higher r^2^value of 0.954 but an equivalent (CV)r^2 ^value of 0.602 from a model containing only 6 components.

The limitations of motif-based methods and the potential of structural analysis may be in part due to the variable nature of the binding with the central region of the peptide. Where as the termini regions (P1-P3 and P8-P9) are heavily constrained by interactions with conserved MHC residues, the central region (P4-P7) typically exhibits a far more variable conformation. Analysis of the MHC Class I structure [1] has shown that the central region is less constrained within the groove than the termini and can dramatically alter its conformation in response to the peptide sequence. It is also the region which is most exposed to the T cell receptor and because of this may arch away from the floor of the groove. The conformational variability of the central region means that definite contacts with specific MHC residues are not obvious. This makes predicting the contribution to affinity of that region of the ligand for the MHC molecule difficult. Nevertheless, the models demonstrate that the central region does make an important contribution to the specificity of the interaction.

It is also possible that static energy analysis overlooks significant energetic interactions occurring during binding. The thermodynamic property, which we are trying to estimate with our scoring function, is the energy released when ligand and receptor bind; the so-called Gibbs free energy of binding: ΔG_bind_. It can be represented as ΔG_bind _= ΔH - TΔS where ΔH is the enthalpic (internal) energy and TΔS is an entropy term which is indicative of the relative gain or loss of disorder upon binding [26]. The binding groove is hydrophobic in character, particularly in the region of the anchor residues, and the calculated energy interactions reflect this, particularly with the high incidence of charge-charge interactions within the groove. It is necessary for the solvent entropy from the burial of hydrophobic groups to offset the reduction in peptide conformational entropy that occurs upon binding. The measured interactions therefore reflect the hydrogen bonds and salt bridges that are formed when the peptide moves from the loosely packed, partially bound state to a being fully bound into a complex. Water is stripped from partial and full charges on the MHC and peptide residues and there is a reduction in the favourable hydrogen bond enthalpy associated from interactions with bound water molecules. In taking our work forward, it is therefore necessary to incorporate the entropic contribution to the energetic calculation into the descriptors in order to create a more accurate representation of the free energy change between the MHC molecules bound and unbound states. This may be done by use of the computationally efficient MM_PBSA/GBSA method which combines the molecular mechanical energies with the continuum solvent approaches [27,28]. It is hoped that the inclusion of these entropic terms may significantly increase the predictive capability of future QSAR analysis. However, the local interactions have always proved to be more relevant to determining the affinity of a given peptide than the general properties of the ligand-receptor interaction [29].

Previous work [10-16] has already indicated that motif-based analysis of the anchor residues within the MHC Class I binding groove can only provide limited accuracy in determining potential T-cell epitopes. Thus the QSAR models presented here strongly challenge the assumption that only the P2 and P9 residues of a nonameric peptide make a significant contribution to binding. While the majority of the descriptors do concentrate on the termini regions of the peptide, there is a clear, and substantial, energetic contribution made by the structurally variable central region. It is possible that more detailed free energy calculations of the MHC Class I-peptide binding interactions, whereby the entropic contribution to the association of the peptide to the MHC Class I molecule is incorporated into the model, will, in the future, lead to the generation of more accurate predictive algorithms.

## Methods

### Energy minimization of HLA-A*0201 structure

An HLA-A*0201 dataset, consisting of 118 peptides of known sequence, was obtained from Doytchinova *et al*. [10]. The structure of the MHC Class I allele HLA-A*0201 bound to a nonameric melanocyte-melanoma tumor-antigen peptide elucidated by X-ray diffraction at a resolution of 2.15Å [2] (*pdb *code: 1JHT) was used as the template. Residues of the bound peptide were remodeled using the crystallographic modeling program 'O' [29] to generate 118 MHC Class I-peptide structures equivalent to the data set. Energy minimization was carried out on the structures using AMBER Version 8 [30]. The interatomic potential energy function uses the AMBER forcefield 03 for parameterisation. Hydrogen atoms were added to the structure, and the system was fully solvated using TIP3 waters [31]. This function was performed by the *LEaP *program [30]. The full structure of the HLA-A*0201-peptide complex was explicitly represented within the simulation. The energy of the solvated molecular complex was minimized using a steepest descent method that continued for 20,000 1 fs time steps or until the root mean square deviation between successive time steps had fallen below 0.01Å. The peptides were then remodeled and annealed by raising the temperature of the system from 0 to 500 K for a period of 8 picoseconds and maintaining the system at that temperature for a further 6 picoseconds. The system was cooled to 0.2 K over a period of 33 picoseconds before being rested at 1 K for a further 30 picoseconds. Both the minimization and the annealing were performed using the *sander *program and static energy analysis was then carried out on the minimised structures using the *anal *program [30]. *Anal *calculates the group-group interaction energies between different parts of the system based upon the position of their composite atoms. The interaction energies, which are measured in kcal mol^-1^, between the nine peptide residues and the first 180 residues of the MHC Class I heavy chain (which incorporates the entire MHC Class I binding groove) were calculated. Three values were generated for each interaction; the electrostatic interaction energy, the van der Waals interaction energy and the total non-bonded interaction energy. Total energies are the sum of electrostatic and van der Waals terms; since these values can have different signs and be significantly different in magnitude, their sum gives rise to a non-trivial and informative descriptor.

### QSAR regression

The BL_50 _binding affinities for the dataset, were extracted from the AntiJen database [32-34]. The half-maximal binding level (BL_50_) is the peptide concentration yielding the half-maximal Fluorescence Index (FI) of the reference peptide in each assay. These values were converted to pBL_50 _(-logBL_50_) and used as the dependent variables in the QSAR regression. Peptides were partitioned between training and test datasets in a ratio of 70:30. A program was developed which transformed the nine amino acid peptide sequence into rows of a table. When choosing a test set, the program omits rows that contain the least contributing amino acid starting at position 1 and working systematically to positions 2, 3, through to position 9 until 30% of the dataset remains. Three different QSAR techniques were employed during the study: the Genetic Function Approximation (GFA) algorithm [35], Genetic Partial Least Squares (G/PLS)[36], which were both carried out using *Cerius*^*2*^[37], and Partial Least Squares (PLS), carried out using *SYBYL 6.9 *[37]. The genetic algorithm first selects equations that contain a randomly chosen subset of dependent variables (or descriptors) and, subsequently, improved equations are 'evolved' using a genetic crossover operation, which selects the best descriptors using multiple linear regression, and finally uses a least squares technique to produce the final model. G/PLS, an alternative to the GFA algorithm, uses GFA to select appropriate basis functions and PLS regression as the fitting technique to weight the basis functions' relative contributions in the final model. In the case of both algorithms, the population size of the initial equation set was fixed at 100 while the number of generations of equations (genetic crossovers) was set at 5000. Once a QSAR calculation has been completed, a list of outliers is generated by the GFA and G/PLS algorithms. Outliers are removed from the training set, the QSAR is repeated again and new equations are generated, until, at most, 10–15% of the training set outliers are removed.

Within *Cerius*^*2 *^the validation terms used were correlation coefficient (r^2^), Friedman's Lack of Fit (LOF)[38] and the Cross-Validation correlation coefficient ((CV)r^2^) using the Leave-One-Out (LOO) procedure [39]. Adding more terms to the regression model always reduces the normal least squares error (LSE) but does not necessarily reduce the LOF measure. Thus, as a new term reduces the LSE, it also tends to increase the value of LOF. Within *SYBYL*, the predictive power of the PLS Cross-Validation Leave-One-Out (*CV-LOO*) model is assessed using the following parameters: cross-validated coefficient (*q*^*2*^) and the Standard Error of Prediction (*SEP*) [10-16]. The optimal number of components (*NC*) resulting from the CV-LOO is then used in the non-cross validated model which was assessed using standard Multiple Linear Regression (MLR) validation terms: explained variance (*r*^*2*^) and Standard Error of Estimate (*SEE*) [10-16]. Increasing the number of components improves the fit between target and explanatory properties and corresponds to the best *q*^*2*^.

## Abbreviations

GFA – Genetic Function Approximation

G/PLS – Genetic Partial Least Squares

MHC – Major Histocompatibility Complex

COMBINE – COMparative BINding Energy

SAC – Superadditivity Correlation

PLS – Partial Least Squares

LOF – Lack of Fit

(CV)r^2 ^– Cross-Validation correlation coefficient

LOO – Leave-One-Out

SEP – Standard Error of Prediction

NC – Number of Components

SEE – Standard Error of Estimate

MLR – Multiple Linear Regression

## Authors' contributions

Matthew Davies remodelled the MHC Class I structures, carried out the energy minimisation simulations and calculated the static energy interactions between the MHC molecule and its bound peptide

Channa Hattotuwagama carried out the QSAR analysis of the static energy interactions data using of Genetic Function Approximation (GFA) and Genetic Partial Least Squares (G/PLS) algorithms

David Moss supervised the molecular modeling work at Birkbeck College

Michael Drew supervised the QSAR analysis at the University of Reading

Darren Flower co-ordinated the analysis of the static energy interactions by QSAR methods and helped to interpret the data in an immunological context as well as overseeing the whole project

## Figures and Tables

**Table 1:** QSAR Results Based on full binding groove for GFA and G/PLS Total, Electrostatic + VDW and Total + Electrostatic + VDW models.

**Model**	**QSAR Analysis** ** (Cerius)**	**Equation Length** ** Terms + Constant**	**STATISTICAL PARAMETERS**						
			
			**Training Set**				**Test Set**		
			
			**LOF**	**r**^**2**^	**(CV) r**^**2**^	**PRESS**	**r**	**r**^**2**^	**PRESS**
**Total + Electrostatic + VDW**	**GFA**	15	0.286	0.766	0.602	22.501	0.466	0.217	46.266
		16	0.340	0.734	0.601	22.538	0.311	0.097	60.902
		**17**	**0.308**	**0.772**	**0.583**	**23.557**	**0.776**	**0.602**	**34.625**
		18	0.311	0.781	0.578	23.840	0.436	0.190	54.676
		19	0.279	0.813	0.663	19.074	0.252	0.063	809.185
		20	0.409	0.741	0.523	26.946	0.031	0.001	74.487
	**G/PLS**	15	-	0.565	0.351	36.678	0.186	0.035	60.451
		16*	**-**	**0.779**	**0.590**	**12.387**	**0.740**	**0.547**	**39.552**
		17	-	0.609	0.167	47.096	0.461	0.213	51.299
		18	-	0.572	0.216	44.329	0.048	0.002	69.939
		19	-	0.620	0.195	45.509	0.155	0.024	64.633
		20	-	0.612	0.113	50.131	0.235	0.055	61.639
**Electrostatic + VDW**	**GFA**	15	0.382	0.687	0.487	29.336	0.087	0.008	68.676
		16	0.316	0.753	0.613	21.905	0.512	0.262	48.315
		17	0.320	0.763	-0.311	74.112	0.514	0.264	59.054
		18	0.351	0.753	-131.834	7510.872	0.525	0.275	43.856
		**19**	**0.349**	**0.767**	**0.616**	**21.719**	**0.731**	**0.534**	**39.464**
		20	0.345	0.781	0.646	20.030	0.523	0.274	39.683
	**G/PLS**	15*	**-**	**0.723**	**0.541**	**21.359**	**0.737**	**0.544**	**52.287**
		16	-	0.681	0.353	36.592	0.760	0.578	37.425
		17	-	0.674	0.303	39.423	0.605	0.366	53.741
		18	-	0.624	-1.892	163.513	0.698	0.488	48.489
		19	-	0.704	0.323	38.271	0.593	0.352	42.913
		20	-	0.686	0.440	31.674	0.650	0.423	40.518
**Total**	**GFA**	15	0.362	0.703	0.252	42.292	0.198	0.039	2866.799
		16	0.349	0.728	0.568	24.420	0.220	0.048	1214.370
		17	0.403	0.701	0.473	29.811	-0.060	0.004	356.214
		18*	**0.200**	**0.861**	**0.751**	**11.071**	**0.781**	**0.610**	**51.105**
		19	0.408	0.727	0.534	26.348	0.487	0.237	65.488
		20	0.388	0.755	0.584	23.517	-0.123	0.015	5580.648
	**G/PLS**	**15**	**-**	**0.665**	**0.299**	**39.635**	**0.796**	**0.634**	**40.751**
		16	-	0.648	0.344	37.116	0.393	0.155	53.478
		17	-	0.646	0.353	36.606	0.375	0.141	55.606
		18	-	0.680	0.131	49.143	0.656	0.430	39.995
		19	-	0.686	0.420	32.816	0.571	0.327	45.867
		20	-	0.662	0.395	34.191	0.253	0.064	61.058

*Indicates where outliers have been removed.

**Table 2:** QSAR Results for GFA Total + Electrostatic + VDW model based on 10, 20 25, 30 descriptors.

**Model**	**QSAR Analysis** ** (Cerius)**	**Equation Length** ** Terms + Constant**	**STATISTICAL PARAMETERS**						
			
			**Training Set**				**Test Set**		
			
			**LOF**	**r**^**2**^	**(CV) r**^**2**^	**PRESS**	**r**	**r**^**2**^	**PRESS**
**Total + Electrostatic + VDW**	**GFA**	10	0.321	0.670	0.562	24.741	0.683	0.467	38.545
		15	0.371	0.696	0.471	29.893	0.752	0.565	27.669
		20	0.288	0.818	0.643	20.164	0.465	0.217	46.796
		25	0.357	0.832	-345.981	19619.436	0.459	0.210	47.097
		30	0.389	0.871	0.688	17.652	0.380	0.144	56.410

**Table 3:** QSAR Results Based on Binding Pockets for GFA and G/PLS Total, Electrostatic + VDW and Total + Electrostatic + VDW models.

**Model**	**QSAR Analysis** ** (Cerius)**	**Equation Length** ** Terms + Constant**	**STATISTICAL PARAMETERS**						
			
			**Training Set**				**Test Set**		
			
			**LOF**	**r**^**2**^	**(CV) r**^**2**^	**PRESS**	**r**	**r**^**2**^	**PRESS**
**Total + Electrostatic + VDW**	**GFA**	10	0.389	0.600	-124.999	7124.376	0.468	0.219	46.369
		11	0.371	0.634	-14.618	883.095	0.510	0.26	55.733
		12	0.362	0.659	-8.917	560.716	0.310	0.096	62.863
		13*	**0.204**	**0.796**	**0.681**	**12.949**	**0.706**	**0.499**	**41.896**
		14	0.349	0.700	-3.314	243.918	0.456	0.208	49.137
		15	0.383	0.686	-4.476	309.647	0.603	0.364	47.871
	**G/PLS**	15	-	0.725	0.378	35.166	0.835	0.697	38.873
		**16**	**-**	**0.789**	**0.611**	**16.029**	**0.843**	**0.711**	**37.423**
		17	-	0.747	0.541	25.978	0.603	0.363	48.927
		18	-	0.751	0.452	30.988	0.300	0.090	73.148
		19	-	0.706	0.210	44.652	0.673	0.453	44.598
		20	-	0.780	-3.724	267.126	0.683	0.566	44.150
**Electrostatic + VDW**	**GFA**	10	0.380	0.609	0.456	30.733	0.484	0.235	54.237
		11	0.382	0.623	-74.601	4105.114	0.711	0.506	43.208
		12	0.381	0.641	-41.045	2377.363	0.539	0.291	51.036
		13*	**0.242**	**0.751**	**0.647**	**15.194**	**0.698**	**0.487**	**37.794**
		14	0.391	0.664	-8.114	515.361	0.550	0.302	45.419
		15	0.376	0.692	-15.787	949.215	0.469	0.220	53.413
	**G/PLS**	15	-	0.572	-569.948	32283.278	0.465	0.217	51.444
		16	-	0.577	-104.575	5969.542	0.511	0.261	48.315
		17	-	0.584	-113.848	6493.914	0.597	0.356	45.638
		18	-	0.593	-122.821	7001.277	0.199	0.039	67.477
		19*	**-**	**0.643**	**0.355**	**23.922**	**0.605**	**0.366**	**40.364**
		20	-	0.601	-47.383	2736.736	0.683	0.466	46.301
**Total**	**GFA**	15	0.436	0.643	-56.544	3253.745	-0.004	0.000	73.957
		16	0.435	0.661	-101.188	5778.054	0.044	0.002	69.087
		17	0.450	0.666	-78.722	4507.744	-0.010	0.000	72.814
		18	0.450	0.683	-65.648	3768.511	0.052	0.003	69.644
		19	0.464	0.690	-89.022	5090.131	0.051	0.003	70.689
		20*	**0.280**	**0.810**	**0.624**	**16.523**	**0.210**	**0.044**	**60.931**
	**G/PLS**	15	-	0.506	-331.296	18789.097	0.103	0.011	66.251
		16	-	0.524	-514.358	29140.068	-0.041	0.002	71.126
		17	-	0.507	-132.796	7565.245	0.016	0.000	73.793
		18	-	0.519	-87.558	5007.347	0.177	0.031	66.411
		19	-	0.514	-359.474	20382.411	0.208	0.043	66.397
		20*	**-**	**0.528**	**0.072**	**28.473**	**0.513**	**0.263**	**58.726**

* Indicates where outliers have been removed.

**Table 4:** Best QSAR models for GFA and G/PLS based on full binding groove in comparison with the PLS method. *PLS Models based on Cerius GFA & G/PLS Models respectively.

**Model**	**QSAR Analysis ^**1**^Cerius ^**2**^SYBYL**	**Equation Length** ** Terms + Constant**	**STATISTICAL PARAMETERS**														
			
			**TRAINING SET**									**TEST SET**					
			
			**LOF**	**SEP**	**r**^**2**^		**(CV) r**^**2**^		**SEE**	**PRESS**		**r**		**r**^**2**^		**PRESS**	
**Total + Electrostatic + VDW**	**GFA**^**1**^	17	0.308		0.772		0.583			23.557		0.776		0.602		34.625	
	**G/PLS**^**1**^	16			0.779		0.590			12.387		0.740		0.547		39.552	
	**PLS^**2 **^(GFA)**	17		0.604		0.630		0.521	0.531		20.526		0.734		0.539		34.247
	**PLS^**2 **^(G/PLS)**	16		0.571		0.615		0.438	0.473		15.869		0.708		0.501		41.424
**Electrostatic + VDW**	**GFA**^**1**^	19	0.349		0.767		0.616			21.719		0.731		0.534		39.464	
	**G/PLS**^**1**^	15			0.723		0.541			21.359		0.737		0.544		52.287	
	**PLS**^**2**^**(GFA)**	19		0.675		0.194		-0.055	0.611		25.038		0.613		0.376		66.115
	**PLS^**2 **^(G/PLS)**	15		0.558		0.669		0.526	0.466		15.863		0.803		0.645		48.045
**Total**	**GFA**^**1**^	18	0.200		0.861		0.751			11.071		0.781		0.610		51.105	
	**G/PLS**^**1**^	15			0.665		0.299			39.635		0.796		0.634		40.751	
	**PLS^**2 **^(GFA)**	18		0.654		0.496		0.350	0.576		23.550		0.777		0.604		58.579
	**PLS^**2 **^(G/PLS)**	15		0.583		0.697		0.537	0.471		16.669		0.861		0.742		35.160

**Table 5:** Best QSAR models for GFA and G/PLS based on binding Pockets in comparison with the PLS method. * PLS Models based on Cerius GFA & G/PLS Models respectively.

**Model**	**QSAR Analysis **^**1**^**Cerius **^**2**^**SYBYL**	**Equation Length ** **Terms + Constant**	**STATISTICAL PARAMETERS**														
			
			**TRAINING SET**									**TEST SET**					
			
			**LOF**	**SEP**	**r**^**2**^		**(CV) r**^**2**^		**SEE**	**PRESS**		**r**		**r**^**2**^		**PRESS**	
**Total + Electrostatic + VDW**	**GFA**^**1**^	13	0.204		0.796		0.681			12.949		0.762		0.499		41.896	
	**G/PLS**^**1**^	16	-		0.789		0.611			16.029		0.843		0.711		37.423	
	**PLS^**2 **^(GFA)**	13		0.593		0.546		0.439	0.533		19.349		0.714		0.510		41.707
	**PLS^**2 **^(G/PLS)**	16		0.704		0.432		0.284	0.628		30.279		0.818		0.699		42.235
**Electrostatic + VDW**	**GFA**^**1**^	13	0.242		0.751		0.647			15.194		0.698		0.487		37.794	
	**G/PLS**^**1**^	19	-		0.643		0.355			23.922		0.605		0.366		40.364	
	**PLS^**2 **^(GFA)**	13		0.548		0.659		0.528	0.466		14.709		0.344		0.118		51.406
	**PLS^**2 **^(G/PLS)**	19		0.729		0.523		0.254	0.583		25.187		0.480		0.230		45.155
**Total**	**GFA**^**1**^	20	0.280		0.810		0.624			-		0.210		0.044		60.931	
	**G/PLS**^**1**^	20	-		0.528		0.072			28.473		0.513		0.263		58.726	
	**PLS^**2 **^(GFA)**	20		0.627		0.539		0.369	0.536		19.527		0.636		0.405		47.833
	**PLS^**2 **^(G/PLS)**	20		0.654		0.434		0.256	0.571		24.467		0.625		0.425		52.133

**Table 6:** Residue descriptors for the Best QSAR Results based on full binding groove.

**Total + Electrostatic + VDW**	**GFA**	Peptide Pos.	P1	P3	P7	P9	P9	P2	P9	P1	P2	P5	P5	P5	P6	P6	P7	P9		
		MHC Residue No.	63	5	141	14	48	39	115	51	69	130	133	144	75	148	140	115		
		MHC Residue	Glu	Met	Gln	Arg	Arg	Asp	Gln	Trp	Ala	Leu	Trp	Lys	Val	Ala	Ala	Gln		
	**G/PLS**	Peptide Pos.	P1	P4	P5	P6	P8	P8	P9	P9	P1	P3	P2	P3	P4	P7	P7			
		MHC Residue No.	171	164	157	66	19	72	77	123	171	156	99	66	66	73	155			
		MHC Residue	Tyr	Cys	Arg	Lys	Glu	Gln	Asp	Tyr	Tyr	Leu	Tyr	Lys	Lys	Thr	Gln			
**Electrostatic + VDW**	**GFA**	Peptide Pos.	P1	P5	P7	P8	P8	P9	P9	P9	P2	P2	P2	P4	P4	P5	P7	P9	P9	P9
		MHC Residue No.	10	35	21	99	117	95	126	153	60	73	160	45	99	130	23	8	117	134
		MHC Residue	Thr	Arg	Arg	Tyr	Ala	Val	Leu	Ala	Trp	Thr	Leu	Met	Tyr	Leu	Ile	Phe	Ala	Thr
	**G/PLS**	Peptide Pos.	P1	P1	P7	P9	P2	P2	P2	P4	P5	P5	P5	P6	P7	P9				
		MHC Residue No.	43	115	22	159	24	68	167	163	116	156	158	152	143	93				
		MHC Residue	Gln	Gln	Phe	Tyr	Ala	Lys	Trp	Thr	Tyr	Leu	Ala	Val	Thr	His				
**Total**	**GFA**	Peptide Pos.	P1	P1	P1	P2	P2	P2	P2	P3	P3	P3	P3	P5	P5	P6	P6	P9	P9	
		MHC Residue No.	25	61	156	33	64	68	163	5	34	162	168	152	156	115	152	77	143	
		MHC Residue	Val	Asp	Leu	Phe	Thr	Lys	Thr	Met	Val	Gly	Leu	Val	Leu	Gln	Val	Asp	Thr	
	**G/PLS**	Peptide Pos.	P1	P2	P2	P2	P3	P3	P3	P5	P6	P7	P9	P9	P9	P9				
		MHC Residue No.	25	33	62	119	5	74	98	156	152	143	14	74	77	83				
		MHC Residue	Val	Phe	Gly	Asp	Met	His	Met	Leu	Val	Thr	Arg	His	Asp	Gly				
